# Supplementary material for: A Potential of New Untreated Bio-Reinforcement from Caesalpinia sappan L. Wood Fiber for Polybutylene Succinate Composite Film
Source: Polymers (Basel). 2022 Jan 26;14(3):499. doi: 10.3390/polym14030499 (PMC8838579; doi:10.3390/polym14030499)
Supplement: Supplementary file 1 [file polymers-14-00499-s001.zip › polymers-1567063-supplementary.pdf]

Supporting Information

A Potential of New Untreated Bio-reinforcement from *Caesalpinia Sappan L.* Fiber for Polybutylene Succinate Composite film

Ekkachai Martwong[1], Yvette Tran[2], Nattawadee Natsrita[3], Chaithip Kaewpang[3], Kittisak Kongsuk[3], Yeampon Nakaramontri[4], and Nathapong Sukhawipat[3,*]

[1] Division of Science, Faculty of Science and Technology, Rajamangala University of Technology Suvarnabhumi, Phra Nakhon Si Ayutthaya, 13000, Thailand

[2] Soft Matter Sciences and Engineering, ESPCI Paris, PSL University, Sorbonne Université, CNRS, F-75005 Paris, France

[3] Division of Polymer Engineering Technology, Department of Mechanical Engineering Technology, College of Industrial Technology, King Mongkut's University of Technology North Bangkok, Bangkok, 10800, Thailand

[4] Sustainable Polymer & Innovative Composite Materials Research Group, Department of Chemistry, Faculty of Science, King Mongkut's University of Technology Thonburi, Bangkok, 10140, Thailand

* Correspondence: Nathapong.s@cit.kmutnb.ac.th; Tel.: (+66)824846930)

Table of Contents

Page

1. FTIR spectra of CSWF, PBS, and PBS/CSWF composite films. 3
2. Thermal degradation characteristics of PBS/CSWF composite film 4
   by TGA and DTG

1. **FTIR spectra of CSWF, PBS, and PBS/CSWF composite films.**

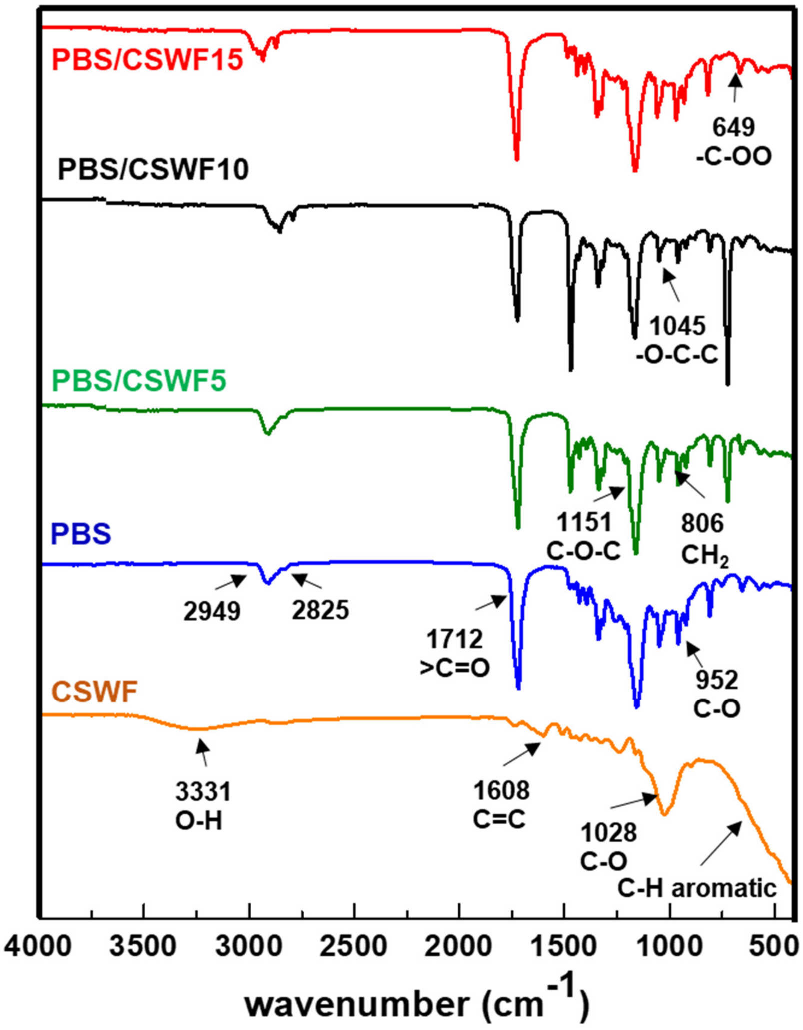

Figure S1. FTIR spectra of CSWF (orange), PBS (bleu), and PBS/CSWF composite films with 5 (green), 10 (black) and 15 (red) phr.

Figure S1 illustrates the FTIR spectra of CSWF, PBS, and PBS/CSWF composite films. The symmetric stretching of the methyl group (– CH3) and the asymmetric stretching of the methylene group (C – H) were found at 2949 and 2825 cm^{-1}, respectively, for neat PBS. The stretching vibration of the ester carbonyl group (>C=O) and C–O–C was observed to exhibit strong peaks at 1712 and 1151 cm^{-1}. The bands at 1045, 952, 806 and 648 cm^{-1} were assigned to O– C– C stretching, C–O stretching, CH_2 in $OC(CH_2)_2CO$ in plane bending, and -COO bending, respectively [1]. CSWF revealed a characteristic peak at 3331, 1608, and 1028 cm^{-1}, which correspond to O–H, C=C, and C–O stretching from cellulose and Brazilin agent in CSWF, as reported in another study [1–3]. The FTIR spectra for the PBS/CSWF composite films were comparable to neat PBS, although there were marked differences.

2. Thermal degradation characteristics of PBS/CSWF composite film by TGA and DTG

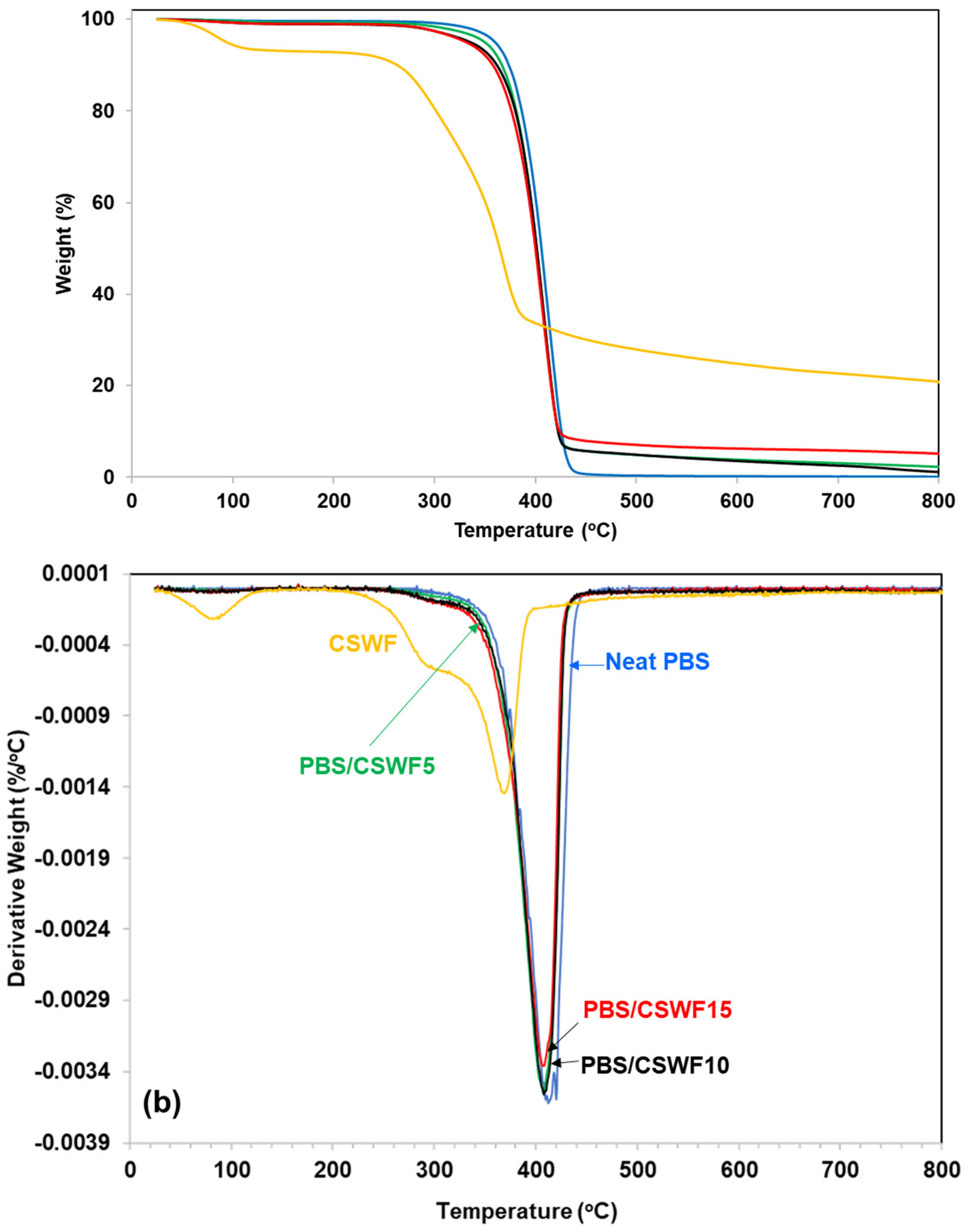

Figure S2. Thermal degradation characteristics of PBS/CSWF composite film by TGA and DTG.

The thermal degradation characteristics of PBS/CSWF composite film series are also investigated using TGA and DTG. As seen in Figure S2, the DTG curves of all PBS composite films exhibit only one distinct step of degradation. The PBS component is

responsible for the most of the degradation. Two step of degradation peaks were observed in the CSWF. The first peak, T_{max} of 85 °C, indicates a moisture loss in CSWF. The second peak, with a T_{max} of 370 °C, represents a cellulose breakdown [4–6]. The degradation temperature of neat PBS film is 413 °C. The thermal stability degradation curves of the PBS/CSWF composite films fall between those of the PBS matrix and the CSWF. The thermal stability of the composites is lowered owing to the incorporation of the CSWF, which is related to other studies [4,7]. T_{max} values decrease slightly as CSWF content increases, indicating 409, 408 and 406 °C for PBS/CSWF5, PBS/CSWF10, and PBS/CSWF15, respectively. Because the breakdown temperature of cellulose is lower than that of PBS, the T_{max} values of composite degradation are slightly reduced. Furthermore, the addition of CSWF enhanced the weight residual, related to the content of additive.

References

1. Muthuraj, R.; Misra, M.; Mohanty, A.K. Injection Molded Sustainable Biocomposites From Poly(butylene succinate) Bioplastic and Perennial Grass. *ACS Sustain. Chem. Eng.* **2015**, *3*, 2767–2776, doi:10.1021/acssuschemeng.5b00646.

2. Ngamwonglumlert, L.; Devahastin, S.; Chiewchan, N.; Raghavan, G.S.V. Color and molecular structure alterations of brazilein extracted from Caesalpinia sappan L. under different pH and heating conditions. *Sci. Rep.* **2020**, *10*, 12386, doi:10.1038/s41598-020-69189-3.

3. Oliveira, L.F.C.; Edwards, H.; Velozo, E.; Nesbitt, M. Vibrational spectroscopic study of brazilin and brazilein, the main constituents of brazilwood from Brazil. *Vib. Spectrosc.* **2002**, *28*, 243–249.

4. Lee, J.M.; Mohd Ishak, Z.A.; Mat Taib, R.; Law, T.T.; Ahmad Thirmizir, M.Z. Mechanical, Thermal and Water Absorption Properties of Kenaf-Fiber-Based Polypropylene and Poly(Butylene Succinate) Composites. *J. Polym. Environ.* **2013**, *21*, 293–302, doi:10.1007/s10924-012-0516-4.

5. Platnieks, O.; Gaidukovs, S.; Barkane, A.; Sereda, A.; Gaidukova, G.; Grase, L.; Thakur, V.K.; Filipova, I.; Fridrihsone, V.; Skute, M.; et al. Bio-Based Poly(butylene succinate)/Microcrystalline Cellulose/Nanofibrillated Cellulose-Based Sustainable Polymer Composites: Thermo-Mechanical and

Biodegradation Studies. *Polym.* 2020, *12*.

6. Mohamad, N.; Mazlan, M.M.; Tawakkal, I.S.M.A.; Talib, R.A.; Kian, L.K.; Jawaid, M. Characterization of Active Polybutylene Succinate Films Filled Essential Oils for Food Packaging Application. *J. Polym. Environ.* **2021**, doi:10.1007/s10924-021-02198-z.

7. Lee, S.-H.; Wang, S. Biodegradable polymers/bamboo fiber biocomposite with bio-based coupling agent. *Compos. Part A Appl. Sci. Manuf.* **2006**, *37*, 80–91, doi:https://doi.org/10.1016/j.compositesa.2005.04.015.
